# PERCEIVED BALANCE IS ASSOCIATED WITH SELF-REPORTED FALLS IN COMMUNITY-DWELLING OLDER ADULTS

**DOI:** 10.1093/geroni/igad104.1955

**Published:** 2023-12-21

**Authors:** Hanne Dolan, Janet Pohl, Keenan Pituch, David Coon

**Affiliations:** Arizona State University, Phoenix, Arizona, United States; Arizona State University, Phoenix, Arizona, United States; Arizona State University, Phoenix, Arizona, United States; Arizona State University, Phoenix, Arizona, United States

## Abstract

Accidental falls are a significant health threat among older adults. Approximately 30% of adults age 65 and older suffer one or more falls each year. Most accidental falls are preventable, and older adults’ engagement in fall prevention is imperative. Limited research suggest that older adults do not use the term “fall risk” to describe their risk for falls. Instead, they commonly use the term “balance problems.” The aim of this study was to examine how perceived balance problems is associated with self-reported falls in the past month after controlling for known predictors of falls among older adults. The Health Belief Model and the concept of perceived susceptibility served as the theoretical framework. A cross-sectional secondary analysis using data from a subsample of independently living participants (N = 7499) from the National Health and Aging Trends Study from year 2015 was conducted, with 10.3% of the sample reporting a fall. Multiple logistic regression analysis revealed that the odds of reporting a fall in the past month were 3.4 times (p < .001) greater for participants who reported having a balance problem compared to those who did not. In contrast, fear of falling and perceived memory problems were not uniquely associated with falls. Using a mobility device, reporting pain, poor self-rated health status, depression, and anxiety scores were also associated with falling. Older adults’ perceived balance problem is strongly associated with their fall risk. A focus on balance instead of fall risk may improve older adults’ engagement in fall prevention.

